# Association between Sleep Disorders and Sleep Quality in Patients with Temporomandibular Joint Osteoarthritis: A Systematic Review

**DOI:** 10.3390/biomedicines10092143

**Published:** 2022-08-31

**Authors:** Eleuterio A. Sánchez Romero, Oliver Martínez-Pozas, María García-González, Miguel de-Pedro, María Elena González-Álvarez, Pablo Esteban-González, Rosana Cid-Verdejo, Jorge Hugo Villafañe

**Affiliations:** 1 Musculoskeletal Pain and Motor Control Research Group, Faculty of Health Sciences, Universidad Europea de Madrid, 28670 Madrid, Spain; 2 Faculty of Biomedical and Health Sciences, Department of Physiotherapy, Universidad Europea de Madrid, 28670 Madrid, Spain; 3 Musculoskeletal Pain and Motor Control Research Group, Faculty of Health Sciences, Universidad Europea de Canarias, 38300 Santa Cruz de Tenerife, Spain; 4 Faculty of Health Sciences, Department of Physiotherapy, Universidad Europea de Canarias, 38300 La Orotava, Tenerife, Canary Islands, Spain; 5 Escuela Internacional de Doctorado, Department of Physical Therapy, Occupational Therapy, Rehabilitation and Physical Medicine, Universidad Rey Juan Carlos, 28933 Alcorcón, Spain; 6 Department of Clinical Dentistry, Faculty of Biomedical Sciences, Universidad Europea de Madrid, 28670 Madrid, Spain; 7 IRCCS Fondazione Don Carlo Gnocchi, 20141 Milan, Italy

**Keywords:** temporomandibular joint disorders, osteoarthritis, sleep wake disorders

## Abstract

Background: Osteoarthritis (OA) is a leading cause of disability, the most common form of chronic disease in the temporomandibular joint (TMJ), and the most severe disease type of temporomandibular disorders (TMD). The etiology of TMD is multifactorial, considering parafunctional habits, sleep bruxism, or sleep disturbance as common factors. Insomnia and apnea are the two most frequent forms of sleep disorders in TMD patients. Due to this, the objective of this systematic review was to highlight whether there is currently scientific evidence in the literature describing that patients with temporomandibular joint osteoarthritis (TMJ-OA) are associated with increased sleep disorders or impaired sleep quality. Methods: This systematic review was completed in accordance with the Preferred Reporting Items for Systematic reviews and Meta-Analysis (PRISMA) statement and was registered with PROSPERO prior to completion of the main search. Original observational studies that analyze the association of sleep disorders and sleep quality in patients with TMJ-OA were included in the present review. Results: 770 studies were screened by abstract and title according to inclusion and exclusion criteria, and finally, 7 articles were included in the qualitative synthesis and a total of 772 patients diagnosed with TMJ-OA. Conclusions: There is insufficient evidence to indicate that patients with TMJ OA are associated with increased sleep disorders or poorer sleep quality.

## 1. Introduction

Osteoarthritis (OA) has a systemic origin and involves an imbalance of factors that promote articular tissue degradation and limit healing, with abnormal tissue metabolism promoting anatomical and/or physiological alterations (such as cartilage degradation, changes in subchondral bone, joint inflammation, or loss of normal function) [1,2]. OA is a leading cause of disability, the most common form of chronic disease in the temporomandibular joint (TMJ), and the most severe disease type of temporomandibular disorders (TMD) [3,4]. TMD are a major problem that affect 5 to 12% population, the most common cause of musculoskeletal pain in the orofacial region [5]. TMD is an umbrella term that encompasses many musculoskeletal problems that include the masticatory muscles, the TMJ, and other associated structures. TMD can be divided into two large groups: those that affect the musculature and those that affect the joint. Within the TMD, we find disk disorders, joint pain, joint disorders, and degenerative joint disease, which includes temporomandibular OA (TMJ-OA) [5,6]. Pain is the most common symptom in TMJ-OA [6]. OA also caused lower levels of synovial fluid that lubricate the joint, affecting the function of the joint negatively [7].

The etiology of TMD is multifactorial, considering parafunctional habits, sleep bruxism, or sleep disorders as common factors [8,9,10]. Sleep disorders have been associated as a risk predictor for developing painful TMD. There is a proportional and bidirectional relationship between the quality of sleep and temporomandibular pain [11,12,13], “pain disturbs sleep, poor sleep exacerbates pain” [14]. TMD affects up to 15% of adults and up to 90% of patients with TMD reported poor sleep quality [15,16]. Insomnia and apnea are the two most frequent forms of sleep disorders in TMD patients [12].

However, pathophysiology of TMD is still unclear. Biopsychosocial factors such as depressive or anxiety symptoms, genes, or sex hormones (sex ratio 2:1 woman: men) have been found higher in TMD patients, and could perpetuate factors of symptoms [14,15]. Also, altered masticatory function or anatomical factors can be also related to its origin [3,6,15]. Sleep bruxism could be also a potential factor to develop a TMD however, there is still a debate in the literature [17]. Chronic disease patients such as migraine, fibromyalgia, or widespread pain also presented painful TMD [6,15]. Furthermore, TMD patients reported lower levels of pain threshold and more sensitivity even in non-cranial sites [14]. Considering that, it is suggested that there is a relationship between these findings and the central/peripheral nociceptive mechanism. Finally, it is not surprising that TMD patients suffer higher disability levels, affecting their quality of life [12].

Due to this, the objective of this systematic review was to highlight whether there is currently scientific evidence in the literature describing that patients with TMJ OA are associated with increased sleep disorders or impaired sleep quality. Further clarification is required to develop better clinical treatment for those patients.

## 2. Materials and Methods

This systematic review was completed in accordance with the Preferred Reporting Items for Systematic reviews and Meta-Analysis (PRISMA) [18] statement and was registered with PROSPERO prior to completion of the main search (Protocol Record Number CRD42022336420).

### 2.1. Focused Question

The aim of the study was to answer the following focused question based on the PRISMA guidelines: Is temporomandibular joint osteoarthritis associated with sleep disorders and sleep quality in patients diagnosed with temporomandibular joint osteoarthritis?

### 2.2. Study Selection

Original observational studies that analyze the association of sleep disorders and sleep quality in patients with TMJ-OA were included in the present review.

The study population included patients diagnosed with TMJ-OA (ICD-9 715.18; ICD-10 M19.91). To be diagnosed, assessment with radiological or computed-tomography scan, as well as via Research Diagnostic Criteria for Temporomandibular Disorders (RDC/TMD) Axis I can be used [19], as well as DC/TMD Axis I protocols [20], but patients need to be classified into “Degenerative Joint Disease”, excluding patients belonging to other groups (I or II).

Sleep quality assessment and sleep disorsders were assessed with questionnaires or neurophysiological studies. Sleep disorsders were based on the *International Classification of Sleep Disorders (3rd edition)* (ICSD), with seven major diagnostics including insomnia, sleep-related breathing disorders, central disorders of hypersomnolence, circadian rhythm sleep-wake disorders, parasomnias, sleep-related movement disorders, and other sleep disorders [21].

### 2.3. Search Strategy

We performed the search for studies on Pubmed, Cochrane Database and Web of Science from inception until 13 May 2022. The main search strategy on Pubmed combined MeSH terms and non-MeSH terms, adding a Boolean operator (OR and/or AND) to combine them. There were no restrictions on language, as recommended by principal guidelines [22].

MeSH terms included “temporomandibular joint”, “temporomandibular joint disorders”, “sleep quality” or “sleep wake disorders”, while non-MeSH included some as “osteoarthritis” or “temporomandibular joint osteoarthritis”. The complete search strategy can be found in Appendix A and the PICO strategy in the following Table 1.

This search string was used on the Pubmed database and modified, if needed, in other consulted databases.

A search strategy was conducted by one independent reviewer (O.M.P) and a reference list of the original studies were screened manually, to identify possible articles. The authors were contacted for further information if necessary.

### 2.4. Selection and Data Extraction

All articles identified from databases were screened by three reviewers (E.A.S.R., O.M.P., M.G.G.). Articles were screened by titles and abstracts to select articles based on the inclusion and exclusion criteria to identify potentially eligible studies. Then, three researchers (E.A.S.R., O.M.P., M.G.G.) independently review the full text of all studies to establish which articles should be included. Any disagreement on the eligibility of studies for inclusion was resolved by consensus.

Data extraction of included studies contains information about sample size, patient status, method of osteoarthritis diagnosis, and sleep assessment. Data was extracted by triplicate independently (E.A.S.R., O.M.P., M.G.G.).

### 2.5. Quality Appraisal

Newcastle-Ottawa Scale (NOS) was used to assess methodological quality, as other authors recommended to evaluate cohort studies [23]. NOS assesses the quality of studies based on three domains: selection (4 items), comparability (1 item), and outcomes (3 items) [24]. “Selection” and “Outcome” domains scores from 0 to 1, and “Comparability” domain score from 0 to 2, with a total score ranging from 0 to 9, with higher scores of better quality. Studies were grouped into good quality (>7/9 points), fair quality (>5–7/9), and low quality (0–4/9), as previous studies did [25].

Three independent reviewers (E.A.S.R., O.M.P., M.G.G.) assessed the risk of bias. In addition, we calculated the kappa coefficient (κ) and the percentage of agreement scores to assess reliability prior to any consensus. Inter-rater reliability was estimated using κ > 0.7 indicating a high level of agreement between the reviewers, κ of 0.5–0.7 indicating a moderate level of agreement, and κ < 0.5 indicating a low level of agreement [26].

### 2.6. Certainty of Evidence

The certainty of the evidence analysis was established by different levels of evidence according to the Grading of Recommendations, Assessment, Development and Evaluation (GRADE) framework, which is based on five domains: study design, imprecision, indirectness, inconsistency, and other considerations [27]. For the risk of bias domain, recommendations were downgraded one level if there was unclear or high risk of bias and severe limitations in the estimation effect. For consistency, recommendations were downgraded when point estimates varied widely among studies, confidence intervals overlapped or when the I2 test was substantial (>50%). For indirectness domain, when serious differences in interventions, populations or outcomes were found, they were downgraded by one level. For the imprecision domain, if there were fewer than 300 participants for key outcomes, it was downgraded one level. Finally, if other considerations were found (as publication bias), one level was downgraded [27].

The evidence was classified into the following four levels: high quality (all five domains are satisfied), moderate quality (one of the five domains is not satisfied), low quality (two of the five domains are not satisfied), or very low quality (three of five domains are not satisfied) [28].

By consensus of the three independent reviewers (E.A.S.R., O.M.P., M.G.G.), the GRADE scale was adapted for domains such as indirectness, inconsistency, imprecision, or publication bias assessment.

## 3. Results

**Figure 1:**
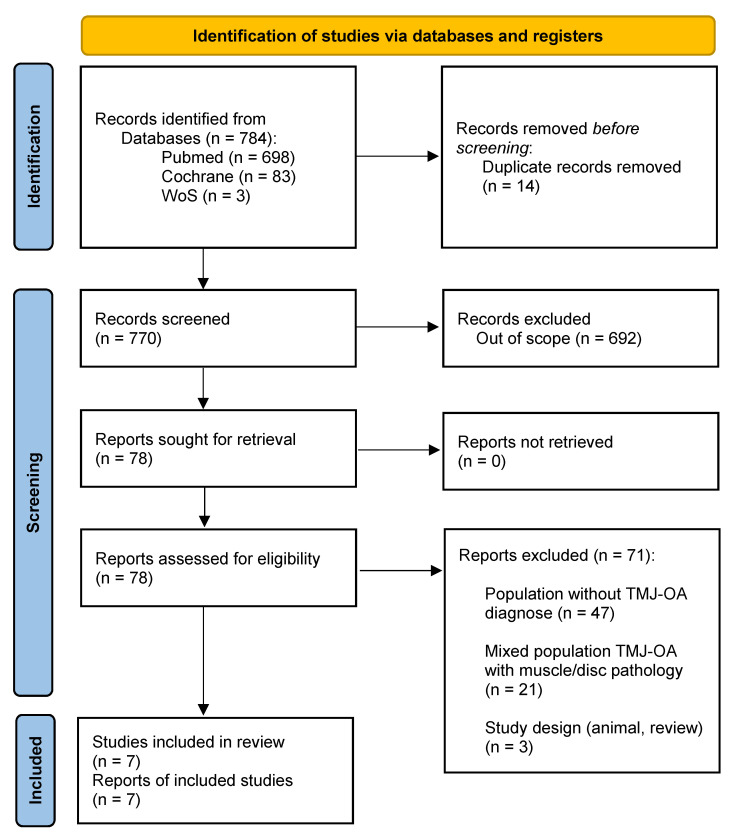
PRISMA flow diagram.

A total of 784 studies were identified through database analysis. After removing duplicates, 770 studies were screened by abstract and title according to inclusion and exclusion criteria. After this, 78 studies were eligible for full text screening. Finally, seven articles were included in the qualitative synthesis [29,30,31,32,33,34,35]. The selection process is shown in Figure 1 (flow diagram).

### 3.1. Characteristic of Included Studies

Of the seven included studies, a total of 772 patients diagnosed with TMJ-OA were included. In all of the studies, the female ratio was higher than the male and was all middle-aged patients (aged between 18 and 85 years old). Table 2 offers an overview of included studies.

According to OA assessment, RDC/TMD Axis was used in isolation in two studies [29,32] cone bean computer-tomography (CBCT) in another study [33], DC/TMD in isolation in one study [34], and radiological assessment in one study [31]. A combination of RDC/TMD and CBCT was used in one study [30], as well as a combination of DC/TMD and CBCT was used in another study [35].

Sleep bruxism (SB) was the most common sleep disorder assessed, mainly measured with ICSD criteria (masseter electromyography) or self-reported questionnaires. Sleep quality (SQ) was evaluated in four of seven studies and Pittsburg Sleep Quality Index (PSQI) was the most common tool to assess it.

### 3.2. Methodological Quality Assessment

Table 3 show the methodological quality of included studies.

Three of the included studies were awarded with 6/9 points (fair quality) [33,34,35], while the four remaining were awarded with 4/9 points (poor quality) [29,30,31,32].

Domains of “Comparability” and “Outcomes” was compromised mostly due to majority of our observational studies did not include control groups and none of them include follow-up periods. The domain of “Selection” obtained the greatest results. Inter-examiner reliability was κ = 0.936.

### 3.3. Quality of Evidence

Quality of evidence was assessed with the Grading of Recommendations, Assessment, Development and Evaluation (GRADE) framework, and results are shown in Table 4.

The GRADE system establishes 4 degrees of evidence (high, moderate, low, and very low), and 2 degrees of recommendation (strong or weak) for or against the intervention; For each item, a judgment is made (very serious, serious, not serious).

Quality of evidence was judged to be low or very low in terms of establishing a relationship between sleep quality or disorders and TMJ-OA, respectively.

Risk of bias, lack of follow-up, lack of control groups, and a low number of studies with low sample sizes were found as mainly downgrading quality items.

### 3.4. Sleep Quality and TMJ-OA

Four studies assessed the relationship between sleep quality and TMJ-OA, with a total of 251 patients analyzed [30,31,33,35].

Three studies found relationships between poor sleep quality and TMJ-OA. They conclude that poor sleep quality was common in patients with TMJ-OA [31,33,35] However, one study found that, although TMJ-OA was presented in patients with poor and good sleep quality, no statistically significant differences between sleep quality and TMJ-OA in patients with temporomandibular Joint disorders (TMJD) [30].

### 3.5. Sleep Disorders and TMJ-OA

Four studies assessed the relationship between sleep disorders and TMJ-OA, with a total of 539 patients analyzed [29,30,32,34].

One study analyzed the relationship between obstructive sleep apnea (OSA) and TMJD, finding that TMJD were present in more than half of the patients in their sample, but TMJ-OA was present only in 12% [29].

Sleep bruxism (SB) was assessed in the remaining three studies. They conclude that, although SB was relatively common in patients with TMJ-OA, no significant associations between SB and TMJ-OA were found [30,32,34].

## 4. Discussion

The main objective of this systematic review was to synthesize the evidence that patients with TMJ-OA are associated with increased sleep disorders or impaired sleep quality.

In terms of the relationship between sleep quality and TMJ-OA, low quality evidence suggests that patients with TMJ-OA usually present lower levels of sleep quality. However, there is no clear relationship between sleep quality and TMJ-OA.

For example, one poor quality study found that in a cohort of 113 adults with TMJD, more than half had sleep quality disturbances [31]. However, sleep quality was assessed with self-reported questions to patients, and validated questionnaires were not used. In the same way, two fair quality studies found that sleep quality was impaired in patients with TMJ-OA and assessed it with the validated Pittsburg Sleep Quality Index [33,35]. Furthermore, Tran Duy et al. found that patients with poor sleep quality are more likely to develop TMJ-OA, with an odds ratio (OR) of 3.64 [33] which is consistent with a recently published cohort study that found that patients with sleep disorders tend to develop OA (OR = 1.25) [36].

In contrast, Dias et al. did not find a relationship between sleep quality and TMJ-OA [30]. However, their sample size was small (n = 18), were limited to females only and methodological quality was poor.

Based on these results, we can conclude that patients with TMJ-OA tend to have lower levels of sleep quality. However, there is no clear relationship between sleep quality and its relationship with TMJ-OA.

Sleep quality and OA are closely related. OA is a leading cause of pain and disability, and increased pain associated with OA during the daytime is related to poor sleep at night which again increases the risk of worsening pain, entering a vicious cycle [37].

A recent systematic review found that patients with poor sleep quality (measured with PSQI) had an OR of 4.45 for developing TMJD, strengthening our results. However, this review includes patients with myofascial pain, joint pathology, or disc displacement problems, so these results should be taken with caution when attending only to TMJ-OA [38].

Additionally, cohorts of patients with hip OA demonstrated that higher levels of pain associated with OA were correlated with higher PSQI scores, indicating poor sleep quality [39]. In addition, higher scores on the Western Ontario and McMaster Universities Osteoarthritis Index (WOMAC) questionnaire, which reflects high disability associated with OA, is considered a predictor of poor sleep quality, highlighting the relationship between functionality and sleep quality [39]. In the same line, Fu et al. found that poor sleep quality (PSQI > 5) was correlated with increased development of fatigue and increased odds of hip pain exacerbations (OR: 1.72 compared to PSQI < 5) in a cohort of 252 patients with hip OA [40].

Regardless of hip OA, similar results are found in other body regions. Fertelli et al. found that in a cohort of 151 patients with knee OA, 64% had poor sleep quality and that difference was statistically significant compared to healthy controls (mean PSQI 6.77 ± 3.02, *p* < 0.05) and even revealed a positive correlation between fatigue and higher scores on PSQI [41].

Although the exact mechanisms of how sleep influences pain and vice versa, sleep problems are related to central sensitization, which may amplify pain in patients with TMJD [42]. Studies on these patients are lacking and more research is needed to evaluate the relationship between TMJ-OA and sleep quality.

On the other hand, when addressing a possible relationship between sleep disturbances and TMJ-OA, included evidence suggests that, although sleep disorders are quite common among patients with TMJ-OA, very low quality of evidence suggests that there seems to be no clear relationship between sleep disorders and TMJ-OA. We believe that the clinical consequences of the entities presented by the patient—in this case TMJ OA- should be the focus of our management and not the entities themselves; beyond the application of preventive treatments in already active degenerative processes, since in many cases patients’ needs are created that do not correspond to their symptomatology: patients with TMJ-OA without consequences at the level of pain, functional limitation, or sleep quality.

Sleep bruxism (SB) was the most common pathology related to TMJ-OA and three included studies analyze their relationship. High heterogeneity was found among the included studies regarding the measurement of sleep bruxism, with one study assessing it with ICSD criteria, another with polysomnography, and the last with Oral Behavior Checklist. The methodological quality of studies was poor [30,32] to fair [34]. Regardless of the assessment of bruxism, none of the included studies found an association between bruxism and degenerative changes in the TMJ. It should be noted that for a definitive diagnosis of sleep bruxism, Polysomnography is the Gold Standard [43].

Regarding obstructive sleep apnea (OSA), one poor quality study found that although TMJD symptoms are quite common in patients with OSA, TMJ-OA was present only in 12% of included sample [29]. However, the included sample was small (n = 32), and their results should be taken with caution. Future studies with these populations should be carried out with larger sample sizes, and also study the groups by OSA severity level.

Looking at other regions of the body, a UK cohort of over 175.000 patients with OA compared to healthy controls revealed that 6.6% of patients had sleep disorders [36]. Furthermore, they found that there was a positive and significant association between sleep disorders and OA (OR 1.25, 95% CI 1.22–1.29). Among sleep disorders, non-organic sleep disorders (OR 1.51, 95% CI 1.44–1.58), hypersomnia (OR 1.79, 95% CI 1.44–2.22) and sleep apnea (OR 3.79, 95% CI 3.20–4.50) were associated with a significantly increased likelihood of developing OA.

Regarding other disorders, OSA is associated with OA in patients with hip or knee OA. In a cohort of patients with hip and knee OA, OSA was prevalent in 66% of patients [44]. It seems that sleep fragmentation due to OSA, leads to increased pain and disability, decreasing the patient’s quality of life. This increased pain impairs normal sleep and establishes a vicious cycle. Another possible relation between OSA and OA is that sleep fragmentation disrupts circadian rhythms, increasing circulating levels of inflammatory cytokines, which may increase catabolism responses and OA progression [45]. However, this positive association between OSA and OA was not found in our results. This could be explained by the small sample size and the lack of studies, as only one study analyzed the prevalence of OSA in TMJ-OA patients. Future studies should investigate this relationship.

Although evidence suggests that sleep disorders are related to knee or hip OA, our results in TMJ-OA do not suggest the same. One possible explanation could be the small number of included studies, as symptomatic TMJ-OA is not investigated the same as other body regions, maybe due to its lower prevalence in the general population. Future studies should investigate whether and how sleep disorders are related to TMJ-OA.

In order to find a relationship between TMJ pathologies and sleep disorders, it is necessary to isolate the object of the study as far as possible and to have homogeneous groups that can be compared with a good control group, in addition to which it is necessary to establish an adequate follow-up period that allows us to follow the evolution in order to reduce the risk of bias.

There are many factors that can affect sleep such as stress, type of work, family life, sedentary lifestyle, obesity, anxiety, possible pathologies present, and the light you receive when you sleep... it is very difficult to separate all these factors to analyze whether TMJ-OA influences sleep and its disorders. The important thing is to create homogeneous groups that have the same factors and the only difference is the presence or absence of the pathology and thus be able to study the relationship in isolation [46,47].

It is necessary to unify the tests used to assess sleep quality, as in this search we have found in the studies by Días et al. [30] and Tran Duy et al. [33], that when using the PSQI they set the cut-off at >5 or, on the other hand, in the study by Yap et al. [35], they set it at >6. According to the study by Ellen Snyder et al. [48], the interpretation of the PSQI scale is on a scale of 1 to 10, with 1 being terrible and 10 being excellent. A score of ≤3 is considered bad, 4 to 6 is considered fair, 7 to 9 is considered good and 10 is considered excellent.

It would be interesting to study the direction of the relationship, meaning whether it is sleep disorders or poor sleep quality that cause the onset of TMJ-OA or conversely whether the onset of TMJ-OA causes subjects to have poor sleep quality and develop sleep disorders.

### Strengths and Limitations

We recognize that our sample of included studies was low, but in return, we decided to perform an analysis of patients diagnosed only with TMJ-OA using inclusion criteria that included only patients diagnosed with TMJ-OA, excluding patients with other joint pathology (arthritis, arthralgia), other intra-articular pathologies (disc pathology) and also excluding patients with myopathy (even if they also had OA) to analyze the pure relationship between sleep quality/disorders and TMJ-OA, since their etiology is very different.

We could not perform a meta-analysis due to the great heterogeneity of the included studies, which should be considered a limitation of the study. However, the systematic review carried out amply responds to the stated objectives.

## 5. Conclusions

Low quality of evidence concludes that TMJ-OA is related to sleep quality. Very low quality of evidence concludes that there is no relationship between TMJ-OA and sleep disorders. It would be interesting to continue carrying out observational studies on this type of subject in a more analytical way and with longer follow-up periods to study whether there are statistically significant relationships.

## Figures and Tables

**Table 1:** PICO strategy used.

PICO Elements	Keywords	Search Items	Search Strategies
**P** (Patient or Population)	Patients diagnosed of TMJ-OA	TMJ-OA	Temporomandibular Joint (MeSH) **OR** Temporomandibular Joint Disorders (MeSH) **OR** Temporomandibular Joint Osteoarthritis **OR** Osteoarthritis
**I** (Intervention) E (Exposure)	Sleep quality and sleep disorders assessed with questionnaires or electronic devices	Sleep quality Sleep disorders	Insomnia **OR** Sleep-related breathing disorders **OR** Central disorders of Hypersomnolence **OR** Circadian Rythm Sleep-Wake disorders **OR** Parasomnia **OR** Sleep-related Movement Disorders **OR** Other Sleep Disorders **OR** Sleep Wake Disorders (MeSH) **OR** Sleep Quality (MeSH)
**C** (Comparison)	Not applicable		
**O** (Outcome)	Relationship between sleep disorders and sleep quality and TMJ-OA		

**Table 2:** Included studies.

Study Name	Study Design	Inclusion Criteria	OA Diagnosis	Sleep Assessment	Results
Cunali 2009 [29]	Cross-sectional study n = 4	Patients with mild-moderate OSA Age: 18–65 y/o	RDC/TMD Axis I	Clinical polysomnography criteria for mild to moderate OSA followed ICSD-2	52% of patients with mild to moderate OSAS present some type of sign and/or symptom of TMD. Prevalence of TMJ-OA in OSA was 12%.
Días 2015 [30]	Cross-sectional study n = 18	Female diagnosed with SB Age: 20–55 y/o	RDC/TMD Axis I + CBCT	SB: ICSD criteria Sleep quality: PSQI	There was no significant association between the pattern of sleep quality (*p* = 0.36), the type of SB (*p* = 0.277), and the presence of degenerative changes of the TMJ. Regardless of the quality of sleep and the type of bruxism presented, the prevalence of degenerative bone disorders was high (67%) among women with a mean age of 46 years and a clinical diagnosis of SB.
Poveda-Roda 2009 [31]	Retrospective cohort study n = 113	Patients with TMJD Age: 13–85 y/o	X-Ray findings + morning stiffness + crepitus	Sleep quality: Questions about sleep difficulties, loss of sleep, awakenings at night.	52.4% of TMJ-OA patients presented sleep disturbances (*p* < 0.05).
Su 2017 [32]	Cross-sectional study n = 515	Patients with TMJ-OA Age: 18–70 y/o	RDC/TMD Axis IIIb	SB: Oral Behavior Checklist Quality of life: OHIP-C14	18.1% of patients with TMJ-OA presented SB. Patients with TMJ OA and SB presented lower quality of life (*p* = 0.012), as well as those with awake bruxism (*p* < 0.001).
Tran Duy 2019 [33]	Prospective cohort study n = 52	Female with Class III maloclussion Age: 18–40 y/o	CBCT	Sleep quality: PSQI	The prevalence of severe TMJ-OA was 74.1% in the poor sleep group and 44% in the good sleep group Participants with poor sleep quality are more likely to develop more DJC-TMJ signs (odds ratio [OR] = 3.64; *p* = 0.027). The relative risk for the study outcome of ≥5 DJC-TMJ signs was 1.68. The poor sleep quality group showed significantly more severe DJC-TMJ (adjusted OR: 5.806; 95% CI: 1.406–23.974; *p* = 0.015) after adjusted for age and severity of chin deviation
Wieckiewicz 2020 [34]	Cross-sectional study n = 2	Adults with TMJD	DC/TMD	SB: Polysomnography (according to the ICSD-3 and ICD-10-CM 27 guidelines based on the electromyographic recording of masseter muscles and audio and video recordings)	No statistically differences in terms of TMJ-OA prevalence among SB and non-SB patients.
Yap 2022 [35]	Cross-sectional study n = 68	Adults with intra-articular TMJD	DC/TMD Axis I + CBCT	Sleep quality: PSQI	Patients with TMJ-OA scored fair-to-poor sleep quality, but without differences compared to patients without intra-articular TMJD.

Abbreviations: SB: Sleep Bruxism; CBCT: Cone-Bean Computed Tomography; ICSD: International Classification of Sleep Disorders; PSQI: Pittsburg Sleep Quality Index; TMJ: Temporomandibular Joint; OA: Osteoarthritis; OR: Odds Ratio; CI: Confidence Interval; OSA: Obstructive Sleep Apnea; TMD: Temporomandibular Disorders; TMJD: Temporomandibular Joint Disorders; RDC/TMD: Research Diagnostic Criteria for Temporomandibular Disorders; DJC-TMJ: Signs of degenerative joint changes in the temporomandibular joints; n: sample size.

**Table 3:** Newcastle-Ottawa Scale for assessing quality appraisal.

Study Name	Selection				Comparability	Outcome			Total
	*1*	*2*	*3*	*4*	*1*	*1*	*2*	*3*	
Cunali et al. 2009 [29]	Y	N	Y	Y	N N	Y	N	N	4
Dias et al. 2015 [30]	Y	N	Y	Y	N N	Y	N	N	4
Poveda-Roda et al. 2009 [31]	Y	N	Y	Y	N N	Y	N	N	4
Su et al. 2017 [32]	Y	N	Y	Y	N N	Y	N	N	4
Tran Duy et al. 2019 [33]	Y	Y	Y	Y	Y N	Y	N	N	6
Wieckiewicz et al. 2020 [34]	Y	Y	Y	Y	Y N	Y	N	N	6
Yap et al. 2022 [35]	Y	Y	Y	Y	Y N	Y	N	N	6

**Table 4:** Summary of findings for clinical trials, including GRADE quality of evidence assessment.

Quality Assessment of Relationship between Sleep Quality and Temporomandibular Joint Osteoarthritis						
**Number of Studies (subjects)**	Risk of Bias	Inconsistency	Indirectness	Imprecision	Other Considerations	Quality
4 (n = 251)	Serious *	Not serious	Not serious	Serious ^†^	None	Low

**Quality Assessment of Relationship between Sleep Disturbances and Temporomandibular Joint Osteoarthritis**						
**Number of Studies (Subjects)**	**Risk of Bias**	**Inconsistency**	**Indirectness**	**Imprecision**	**Other** **Considerations**	**Quality**
4 (n = 539)	Serious *^,‡^	Serious ^±^	Not serious	Serious †	None	Very Low

* Lack of follow-up, ^‡^ No control group, ^±^ Mixed results, ^†^ Low number of studies/subjects or size effects not reported.

## Data Availability

The data presented in this study are available on request from the corresponding authors.

## Appendix A. Search String

**Search string (Pubmed)**

((Temporomandibular Joint[MeSH] OR Temporomandibular Joint Disorders[MeSH] OR (Temporomandibular joint osteoarthritis OR Temporomandibular joint disorders OR osteoarthritis) AND (Insomnia OR Sleep-related breathing disorders OR Central disorders of hypersomnolence OR Circadian rhythm sleep-wake disorders OR Parasomnia OR Sleep-related movement disorders OR Other sleep disorders OR Sleep Wake Disorders[MeSH] OR Sleep Quality[MeSH]) NOT (“review”[TIAB]) NOT (“Meta-Analysis”[TIAB]) NOT “Case Reports”[TIAB]))

N = 698 (13/05/2022). 6 Included.

**Search string (Cochrane Database)**

(Temporomandibular Joint OR Temporomandibular Joint Disorders OR Temporomandibular Joint Osteoarthritis) in Title Abstract Keyword AND (Insomnia OR Sleep-related breathing disorders OR Central disorders of hypersomnolence OR Circadian rhythm sleep-wake disorders OR Parasomnia OR Sleep-related movement disorders OR Other sleep disorders OR Sleep wake disorders OR sleep quality)

N = 83 (13/05/2022) results. 0 included.

**Web of Science**

(AB = ((Temporomandibular Joint OR Temporomandibular Joint Disorders OR Temporomandibular joint osteoarthritis OR Temporomandibular joint disorders)) AND AB = ((Insomnia OR Sleep-related breathing disorders OR Central disorders of hypersomnolence OR Circadian rhythm sleep-wake disorders OR Parasomnia OR Sleep-related movement disorders OR Other sleep disorders OR Sleep Wake Disorders OR Sleep Quality))) AND TI = (Osteoarthritis)

N = 3 (13/05/2022). Included: 1.
